# A Model of Predictive Postural Control Against Floor Tilting in Rats

**DOI:** 10.3389/fnsys.2021.785366

**Published:** 2021-11-25

**Authors:** Akira Konosu, Tetsuro Funato, Yuma Matsuki, Akihiro Fujita, Ryutaro Sakai, Dai Yanagihara

**Affiliations:** ^1^Department of Life Sciences, The University of Tokyo, Meguro-ku, Japan; ^2^Department of Mechanical Engineering and Intelligent Systems, The University of Electro-Communications, Chofu, Japan

**Keywords:** predictive postural control, bipedal rats, motor learning, model predictive control, simulation

## Abstract

Humans and animals learn the internal model of bodies and environments from their experience and stabilize posture against disturbances based on the predicted future states according to the internal model. We evaluated the mechanism of predictive control during standing, by using rats to construct a novel experimental system and comparing their behaviors with a mathematical model. In the experiments, rats (*n* = 6) that were standing upright using their hindlimbs were given a sensory input of light, after a certain period, the floor under them tilted backward. Initially, this disturbance induced a large postural response, including backward rotation of the center-of-mass angle and hindlimb segments. However, the rats gradually adjusted to the disturbance after experiencing 70 sequential trials, and a reduction in the amplitude of postural response was noted. We simulated the postural control of the rats under disturbance using an inverted pendulum model and model predictive control (MPC). MPC is a control method for predicting the future state using an internal model of the control target. It provides control inputs that optimize the predicted future states. Identification of the predictive and physiological parameters so that the simulation corresponds to the experiment, resulted in a value of predictive horizon (0.96 s) close to the interval time in the experiment (0.9–1.15 s). These results suggest that the rats predict posture dynamics under disturbance based on the timing of the sensory input and that the central nervous system provides plasticity mechanisms to acquire the internal model for MPC.

## Introduction

Most daily activities and skillful motor performance require predictive postural control to stabilize the posture against internal and external disturbances. Predictive postural controls are based on neuroplasticity, which enables learning of the internal models, namely, the relationship between motor output and the resulting posture changes in a certain environment. Although a wide range of nervous system areas, centered in the cerebellum, have been suggested to be involved in predictive postural control (Diener et al., 1992; Yakovenko et al., 2011; Ng et al., 2013; Yanagihara, 2014; Bolzoni et al., 2015), the detailed mechanisms remain unclear.

In humans, one of the most important and challenging tasks in postural control is to maintain an upright standing posture against gravity. Disturbance systems, in which a floor tilts backward while participants maintain a static standing posture, greatly contribute to understanding the stabilization mechanisms of the upright posture and finding automatic responses with a latency of approximately 100 ms (Nashner, 1976; Carpenter et al., 1999). Several studies have examined predictive postural controls for external disturbances while incorporating classical conditioning into floor-tilting systems. In the association between floor tiles and the preceding sensory inputs, muscle activities around the leg joints and center of pressure (CoP) started to fluctuate just before the tilt (Kolb et al., 2002). In addition, even without the floor tilt, the sensory input alone evoked predictive movement to cancel the upcoming postural response and suppress the excessive stretch reflex as a result of this association (Campbell et al., 2009). Furthermore, it has been found that patients with cerebellar defects cannot establish these predictive controls (Kolb et al., 2004).

Neuroscience has evolved as a result of powerful research methods, these include recording neural activities, genetic manipulation, and temporal and/or region-specific inactivation in rodents. Developing experimental systems for predictive postural control in rodents would greatly advance the understanding of these mechanisms. We have developed a system for evaluating predictive postural controls for voluntary movements has been developed in mice (Yamaura et al., 2013). Recent advances in the identification of the genetic basis of hereditary cerebellar ataxias have made it possible to produce mouse models for these diseases. Spinocerebellar ataxia is a type of cerebellar disease associated with an autosomal dominant pattern of inheritance. The most common type is spinocerebellar ataxia type 3, also known as Machado-Joseph disease. We generated a conditional transgenic mouse model with spinocerebellar ataxia type 3 mice, which have defective cerebellar Purkinje cells due to the induced expression of the Purkinje cell-specific L7 promoter (Yamaura et al., 2013). In the voluntary reaching task, that performed dorsiflexion of the neck as the prime movement, to reach and drink from a water flask while standing, spinocerebellar ataxia type 3 mice showed postural deficits that are characterized by considerable variation in the trajectory of the mouth, terrible swaying posture, and delayed electromyography activities in the hindlimb muscles. This study demonstrated that predictive postural controls can be kinematically and physiologically evaluated in rodents. However, to our knowledge, an experimental system to evaluate predictive controls against external disturbances has not yet been established. To understand the neural basis of it, applying the systems of upright standing and floor tilting in rodents could be effective. We have previously established an experimental environment in which rats maintained an upright posture using hindlimbs on a static floor, confirming that the intersegmental coordination and frequency characteristics of the CoP are consistent with those of humans (Funato et al., 2017). In the present study, we establish a novel experimental task in rats by incorporating a floor tilting disturbance and a conditioning paradigm into this system.

To approach the control mechanism for predictive behavior, we used a mathematical model of postural control. In previous studies, we developed a postural control model for bipedally standing rats with lesions in the olivo-cerebellar system (Funato et al., 2021), and showed that the decreased non-linear control, possibly due to lesions in the internal model, caused instability. The current experiment conducted on rats is based on this study, thereby the mathematical model is also based on this study. The same body model of rats can be used, and an additional component for predictive behavior is needed. To model the predictive behavior of rats, a mathematical model of postural control requires prediction control. One such control system is the model predictive control (MPC). MPC predicts the future state using an internal model of the control target and determines the control inputs so that the predicted state becomes optimal. MPC has been used for the gait and postural control of humanoid robots (Alcaraz-Jiménez et al., 2013; Scianca et al., 2020) and has been used as a model for human quiet standing (Yao and Levine, 2009), walking (Sun et al., 2018), and arm movement during standing (Shen et al., 2021). Prediction time, which is the extent to which future behavior is predicted for control, is a major parameter for determining the behavior by MPC. By comparing the behavior of the mathematical model with the simulation and the behavior of rats in the experiment, we can evaluate the prediction time of rats in the experiment. A comparison of the estimated prediction time of the rats and the experimental conditions will show the control characteristics of the prediction behavior in rats.

In this study, we numerically evaluated the neural mechanism of predictive postural controls against external disturbances through a combination of experiments in rats and simulation with MPC.

## Materials and Methods

### Experimental Animals

Six Wistar rats (male, 19 ± 2 weeks old, 404 ± 28 g body weight) supplied by CLEA (Tokyo, Japan) were used for this experiment. They were kept in a room with a constant temperature and a light and dark cycle of 12 h, with access to food and water *ad libitum*. The experiment was approved by the Ethical Committee for Animal Experiments at the University of Tokyo and was conducted in accordance with the Guidelines for Research with Experimental Animals of the University of Tokyo.

### Experimental Protocols

**FIGURE 1:**
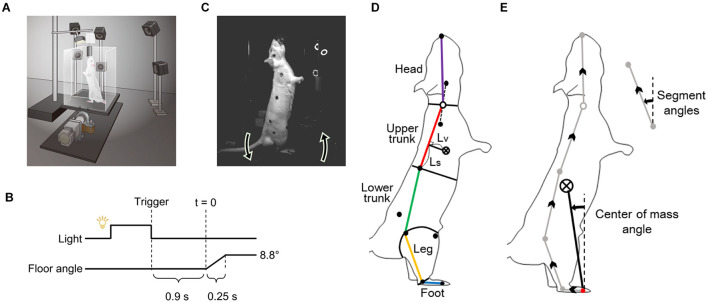
Experimental methods. **(A)** Experimental apparatus. **(B)** Paradigm of the floor tilting trial. **(C)** A rat maintaining upright posture and rotation direction of the floor. **(D)** Definition of body segments. **(E)** Definitions of center-of-mass angle and segment angles.

Postural tasks were performed on a custom-made rotating floor in a dark room (Figure 1A). The rats were habituated to the experimental environment and learned to stand upright using hindlimbs. This was followed by 70 sequential tilting trials performed during 1 day. The paradigm of one trial was as follows (Figure 1B). First, the lights installed in front of the rat were turned on, simultaneously, 4% sucrose water was supplied as a reward through a flexible tube suspended above the center of the floor and accumulated at the tip. The rat stood upright on the hindlimbs in the direction of light to drink the water. The measurer entered an electrical trigger into the experimental system after confirming that the rat was in the proper posture (the body was stationary and the stomach was facing the light). This trigger turned the light off, and the floor began to rotate backward (toes-up, Figure 1C) about 0.9 (0.91 ± 0.04) s later. This interval time was within the range in which classical conditioning can be established (Kolb et al., 2002). The floor rotated by 8.8 degrees over 0.25 s, with a constant angular velocity.

The height of the water supply port was determined during the habituation period for each rat, to ensure that the heel did not float and the hip did not touch the floor in the upright position (24.0 ± 0.3 cm).

### Measurement of Kinematics

Prior to the measurements, the right-sided body surface of the rats was shaved, and markers using black ink were applied to eight body landmarks [4th metatarsophalangeal (MTP) joint, lateral malleolus, knee joint, greater trochanter, iliac crest, scapula, the midpoint between the iliac crest and scapula, and the temporomandibular joint] under anesthesia with 2.5% isoflurane gas. Six high-speed cameras (Prime 13 and 13 W, NaturalPoint Inc., United States) recorded the movements of the right side of their body, at 200 Hz in infrared mode, after the trigger.

### Motion Analysis

The two-dimensional coordinates of nine markers (the above eight markers and nose, black points in Figure 1D) on the image were calculated using DeepLabCut, an image analysis software based on deep learning (Mathis et al., 2018), for the video of each camera in the trials. The three-dimensional coordinates of each marker were calculated using the direct linear transformation method (Abdel-Aziz and Karara, 2015). The coordinates were then smoothed with a 15 Hz 4th-order Butterworth low-pass filter. Sagittal plane motion analyses were performed using a 5-segment rigid link model consisting of the foot, leg, lower trunk, upper trunk, and head segments (Figure 1D). The inertial parameters of the segments were determined using the following procedure. First, a rat was frozen in the upright position with the markers applied as in the experiment and dismantled into the segments. Then, for each segment, the mass was measured, and the center of mass (CoM) was determined as a fulcrum, balancing the segment when it was hung with a string. These procedures were performed on three individuals, including those used for the experiment (440 ± 28 g body weight), and an average of the parameters was used for the analysis (Table 1).

**TABLE 1:** Inertial parameters of the segments.

**Segments**	**Mass (%)**	**Ls (%)**	**Lv (%)**
Foot	1	32	−
Leg	9	68	−
Lower trunk	53	18	33
Upper trunk	29	40	10
Head	8	33	−7

*The masses are shown as percentages of the whole body, and those of the foot and leg segments are the sum of both hindlimbs. As shown in Figure 1D, the center of mass of each segment is shown as coordinates in the coordinate systems formed by the direction of the segment line and the direction orthogonal to it and as the percentages of the line lengths (Ls and Lv, respectively). Because the foot and leg segments were linear in shape and too small to hang with a string, the Lv was assumed to be 0.*

For each trial, the CoM angle (the angle of the vector from the MTP joint to the whole-body CoM) and the angles and angular velocities of the segments were calculated. These parameters are shown as the angles with the perpendicular line and backward rotation (Figure 1E).

In addition, the effects of segment rotations on the CoM angle (*E_i*) were estimated using the following formula:


(1) $$E_{i}=\int_{0}^{0.3}dt\,\frac{m_{i}}{M}\cdot \frac{\text{r}\,\left(t\right)\cdot {\text{r}}_{i}\,\left(t\right)}{\left|\text{r}\,\left(t\right)\right|\,\left|{\text{r}}_{i}\,\left(t\right)\right|}\,\omega_{i}\,\left(t\right)$$

where *i* is the segment of interest, *m*_*i*_ is the mass of the body part above the segment *i* (e.g., collection of the leg, lower trunk, upper trunk, and head segments when *i* is the leg segment), *M* is the mass of the whole body,**r**(**t**) is the vector from the MTP joint to the whole body CoM, **r_i_**(**t**) is the vector from the lower end marker of the segment *i* (e.g., lateral malleolus when *i* is the leg segment) to the CoM of the body part above segment *i*, and ω_*i*_(*t*) is the angular velocity of segment *i*. The integrand of this equation was calculated for each frame based on the experimental data, and a discrete-time integration was performed for the interval from the start of floor tilt to 0.3 s later. It was assumed that the body part above the segment of interest was a rigid body and rotated around the lower end of the segment.

### Model Predictive Control Model and Simulation

**FIGURE 2:**
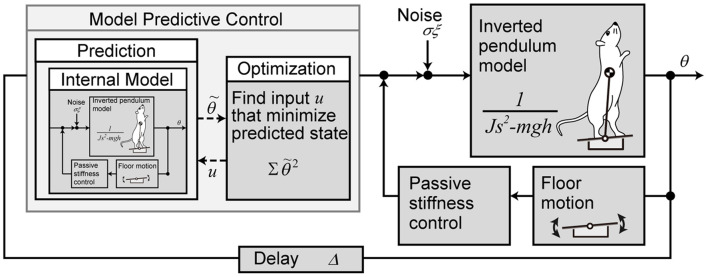
Mathematical model of the postural motion of a rat with the prediction for tilting disturbance. The body is modeled with a single link inverted pendulum, and the control system is modeled with model predictive control.

We used a mathematical model to reproduce the behavior of the rats to control with the prediction of future disturbances after a light stimulus. A block diagram of the mathematical model is presented in Figure 2.

The body of the rat was modeled as an inverted pendulum with one link from the MTP joint to the CoM.


(2) $$J\,\ddot{\theta }=m\,g\,h\,\sin\theta +\tau +\tau_{F\,l\,o\,o\,r}+\sigma \,\xi$$

where θ is the angle of the body from the vertical (elevation angle), *J* is the moment of inertia, and *m*, *h*, and *g* are the mass of the rat, the length from the MTP joint to the CoM, and the acceleration of gravity, respectively. The control torque is τ, the torque due to biological noise is σξ, where time-variant function ξ(t) is a Gaussian white noise and time-invariant coefficient σ is the intensity (Asai et al., 2009). τ_*Floor*_ is the torque input to the body according to the floor inclination and is explained below.

The stability of the rat’s body is partially supported by the physical stiffness of the muscles around the MTP joint during standing (Winter et al., 1998). The torque generated at this time is modeled by the elasticity *k_P* and viscosity *k_D* of the muscles around the MTP joint.


(3) $$\tau_{F\,l\,o\,o\,r}=-k_{P}\,\left(\theta -\phi \right)-k_{D}\,\left(\dot{\theta }-\dot{\phi }\right)$$

where ϕ is the angle of the floor from the horizontal line. In the stationary standing position, it is zero and acts to stabilize, whereas when the floor is inclined, τ_*Floor*_ acts as a disturbance torque according to the inclination.

The control system with prediction was modeled with MPC. MPC predicts the state from the current time to *H_p* steps later, based on the internal model. It also derives the control inputs that optimize the predicted states by providing control inputs with different values for *H_u* steps. MPC performs this prediction and optimization in each time step and uses the input of only the first step as the actual control input. Here, *H_p* is called prediction horizon, and *H_u* is called control horizon. In our model, the inverted pendulum model (Eq. 2) and the floor model (Eq. 3) are used as the internal model for prediction, assuming that the internal model has been created accurately by sufficient learning. The target value of the predicted state $\tilde{\theta }$ for the state θ, is always set to zero. For optimization, an evaluation function is set to minimize the sum of the squares of $\tilde{\theta }$ for the *H_p* step interval.

The proposed model has five unknown parameters other than the parameters determined by the physical properties of the rat’s body: the prediction horizon *H_p*, the control horizon *H_u*, the elastic and viscous coefficients *k_P* and *k_D* around the MTP joint, and the noise magnitude σ. By simulating the mathematical model, we investigated the effects of these parameters on the behavior of the model. In the simulation, we used the average values of the body parameters of the rats (*n* = 6) used in the experiment (m = 0.404 kg, h = 0.107 m). The sensory delay was set to 40 ms based on a previous study on rats (Muramatsu et al., 2009). The sampling time of the simulation and MPC is set to 0.001 s. Model Predictive Control Toolbox of Matlab/Simulnk is used to implement the MPC.

To compare the behavior of proposed model with conventional feedback control models, we further simulated the conventional model with floor disturbance using Eq. (3). The control models included the PD controller based on the Peterka’s gains [$\tau =-k_{P}\,\theta -k_{D}\,\dot{\theta }$, *k*_*P*_ = 1.46*mgh*, *k*_*D*_=0.3*mgh*; (Peterka, 2002)], and the non-linear PD controller for standing rats [$\tau =-k_{P\,2}\,\theta^{2}-k_{P\,0}\,\theta -k_{D}\,\dot{\theta }$, *k*_*P*2_=196*mgh*, *k*_*P*0_=0.88*mgh*, *k*_*D*_=0.11*mgh*; (Funato et al., 2021)]. Here, the control gains were normalized with *mgh* to eliminate differences in the body.

### Quantitative Evaluation of the Rats’ Behavior From the Mathematical Model

We compared the behavior of the constructed mathematical model with that of the rat and examined the characteristics of the controller that reproduces the behavior of the rat. For this purpose, we identified the unknown parameters (prediction horizon *H_p*, control horizon *H_u*, elastic coefficient around the MTP joint *k_P*, viscous coefficient *k_D*, and noise magnitude σ) of the mathematical model that best reproduced the behavior of the rat.

To identify the parameters, we compared the time series of the CoM angle from the experiment with that of the body angle θ from the simulation, and searched for the parameters that minimize the squared error. Specifically, the time sequences of CoM angle from −0.35 s to 0.45 s (here, 0 s is the start of floor tilt) of the terminal 3 trials in each rat are extracted (18 in total), and are compared with the simulation for identification. To reduce the effect of noise variation in the parameter search, the simulation was repeated for five trials and averaged for each evaluation. We used a genetic algorithm (GA) for parameter searching. The “ga” function of Matlab Global Optimization Toolbox was used for GA. In summary, MPC searches were conducted to identify the optimal control input in each simulation step (every 0.001 ms), and simulation with real-time optimal input was repeated five times with same parameters. Then, the average of the simulation result was compared with the experiment for updating the parameter values once. Subsequently, the next simulation with real-time optimization was started for the next update of parameters. The parameters were identified by repeating these sequences using the GA.

### Statistical Analysis

Trials in which the rats lifted their feet off the floor during the tilt were excluded from the analyses. Parameters calculated based on marker coordinates that may not have been calculated accurately by DeepLabCut (“likelihood”<0.9) were also excluded. The results of the analysis are shown as the mean ± standard deviation of the six rats. For the displacements of CoM and segment angles from the start of the tilt to 0.3 s later, the differences in the average of the initial and terminal eight trials were tested with the paired *t*-test using SPSS (IBM, United States), in which *p* < 0.05 was considered as significant.

By comparing the experimental results and the simulation results of the proposed model, the values for five unknown parameters were identified. To validate the significance of the identification results, we used a two-way ANOVA with experimental sequences and parameters.

## Results

### Experimental Results

**FIGURE 3:**
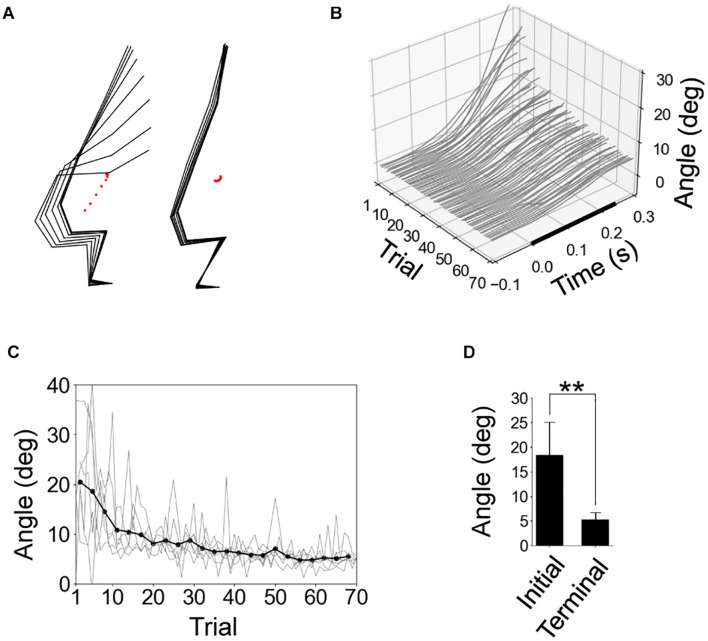
Reduction of postural response due to experience of tilting trials. **(A)** Stick pictures of first and last trials in a representative rat. The period from the start of floor tilt to 0.28 s later was shown using black-point markers in Figure 1C. **(B)**. Time series of center-of-mass (CoM) angles of all 70 trials in a representative individual, with the start time of the tilting at time 0. **(C)** Fluctuation in CoM angle from the start of tilt to 0.3 s later for all individuals (gray line; *n* = 6) and the average (black line, the averages of 3 peripheral trials). **(D)** Statistical comparison of fluctuation in CoM angle from the start of tilt to 0.3 s between the average of initial and terminal eight trials by paired *t*-test (*n* = 6). **P < 0.01.

In the initial trials, the rats experienced a significant response of the whole body due to the floor tilt, but the amplitude of this response was reduced in the later trials (Figure 3A). Figure 3B shows the time series of the CoM angles of all trials in a representative individual. The CoM angle monotonically increased after the start of the tilt, but this fluctuation gradually decreased with repeated trials. We quantified the increase in the CoM angle from the start of the tilt to 0.3 s (Figures 3C,D). The increase was significantly smaller in the terminal eight trials than in the initial eight trials. The difference in the increase between the first and 11th trial (9.9 deg in average) was larger than the difference between the 11th and final trial (3.6 deg in average), suggesting that the rats primarily learned to compensate for the floor tilt in the initial 10 trials.

**FIGURE 4:**
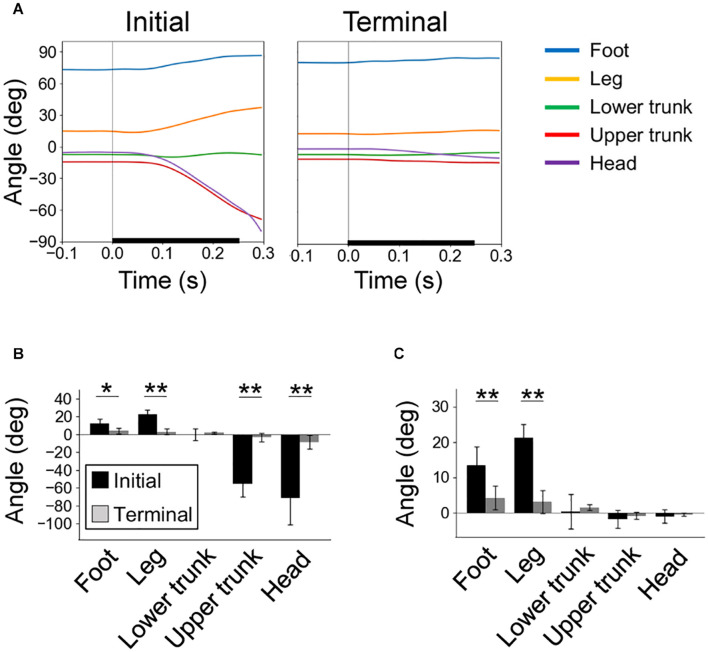
Rotation of body segments due to perturbation. **(A)** Time series of segment angles in the initial (left) and terminal (right) trials, with the start time of floor tilting at time 0. Definitions of segment angles are in Figure 1E. **(B)** Rotation angles of segments from the start of floor tilt to 0.3 s later. **(C)** Effects of segment rotations on the CoM angle calculated by Eq. (1). In **(A–C)**, individual averages (*n* = 6) of trial averages (initial and terminal eight) are shown. *P < 0.05; **P < 0.01.

In the initial trials, the foot and leg segments rotated backward along with the backward tilt of the floor, whereas the upper trunk and head segments rotated forward (Figures 4A,B). These rotations decreased significantly during the trials. We estimated the effects of segment rotation on the CoM angle using Eq. (1). It was found that, in the initial trials, the backward rotations of the foot and leg segments largely affected the increase in the CoM angle after the start of floor tilts (Figure 4C).

### Simulation of the Proposed Model

**FIGURE 5:**
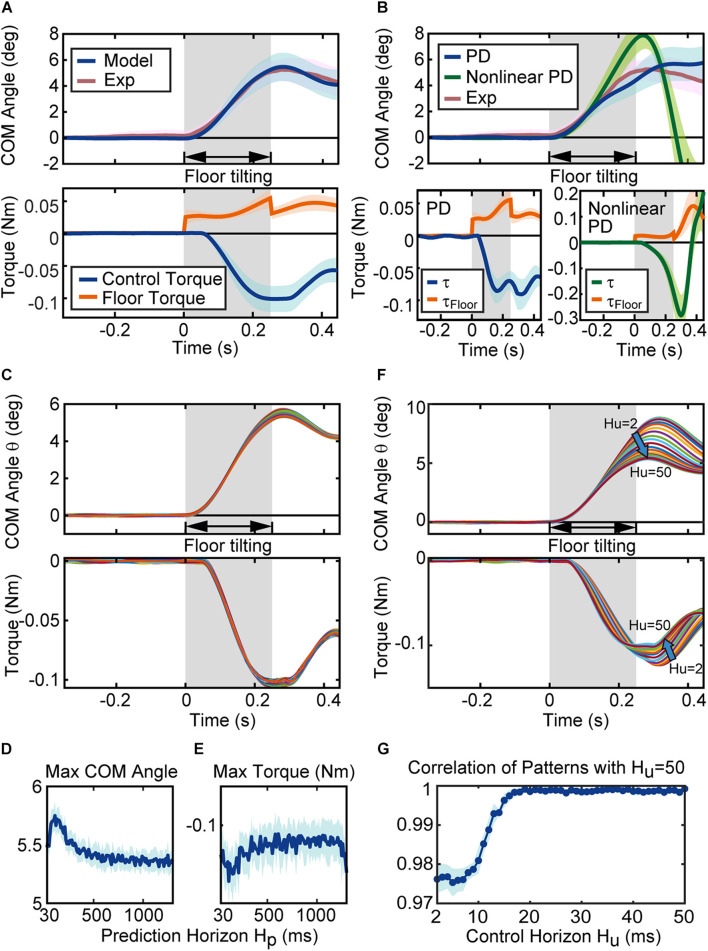
Behavior of the proposed mathematical model. **(A)** Simulation results of the model. The blue and red lines in the figure of CoM angle are the average of the CoM angles for the 17 simulated (Model) and experimental (Exp) sequences. The blue and red regions represent their standard deviation (SD). The blue and orange line in the torque figure represent the average of the control torque τ and the floor torque τ_*Floor*_, respectively. The surrounding blue and orange regions represent the associated SD. **(B)** Simulation results of the conventional models. Blue lines, green lines and red lines in the figure of CoM angle are the average results of the PD controller with Peterka’s gain parameters, the average results of the non-linear PD controller for standing rats, and the average of the experimental sequences, respectively. Blue, green, red regions represent their SD. The blue, green, and orange line in the figure of torque represent the average of the control torque τ and the floor torque τ_*Floor*_, respectively. The blue, green and orange regions represent their SD. **(C)** Time series of the CoM angle and torque with different prediction horizons. Each line in the figure is the simulation result of the model with a prediction horizon from 30 ms to 1,300 ms (average of ten simulation results with same parameters). The maximum CoM angle and the maximum torque for each parameter are shown in **(D,E)**, respectively. **(D,E)** Are the results for one sequence, and Supplementary Figure 2 shows the results for complete sequences. **(F)** Time series of the CoM angle and torque with the control horizon from 2 ms to 50 ms. Each line is the average of ten simulations with the same prediction horizon. **(G)** The correlation coefficient (cosine correlation) between the time series of the CoM angle (average of ten trials) with control horizon 50 ms and the time series of the CoM angle with each control horizon. The line is the average of ten correlation coefficients for each control horizon, and the area represents SD. **(G)** Is the results for one sequence, and Supplementary Figure 2 shows the results for complete sequences.

**FIGURE 6:**
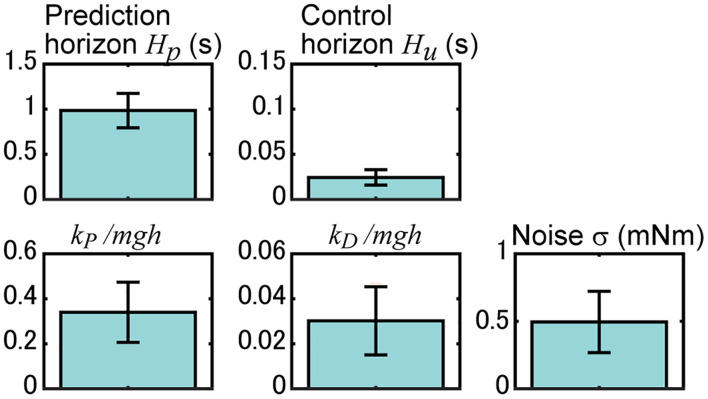
Identified parameters of the proposed model. Results for the prediction horizon *H_p*, control horizon *H_u*, MTP stiffness *k_P*, MTP viscosity *k_D*, and noise magnitude σ are shown. Here, the results of MTP stiffness and viscosity are normalized with *m* body mass, *g* acceleration of gravity, and *h* length from MTP joint to CoM. Blue bars and their error bars represent the average and standard deviation of the identified results for 18 experimental sequences of rats.

To approach the mechanism of the rats’ predictive control for tilt disturbance, we constructed a mathematical model using MPC and investigated the behavior of the model by simulation. Figure 5A shows the results of the CoM angle and torque in the simulation. The blue line in the CoM angle of Figure 5A is the result of the simulation, and the red line shows the rats’ CoM angle from the experiment (the average of 17 sequences out of the measured 18 sequences, excluding one sequence in which the rat’s behavior was different from the other sequences [Rat 5 Sequence 1]; a comparison of the results for each sequence is shown in Supplementary Figure 1). The unknown parameters of the mathematical model used in the simulation were set to the optimal values (Figure 6), which will be described later in detail. The mean (SD) of the correlation coefficient (cosine correlation) between the CoM angle from the simulation and from the experiment for all the 18 sequences were 0.99 (± 0.01, Supplementary Figure 1 shows the correlation coefficient for each sequence). These results show that the proposed system using MPC successfully reproduced the behavior of the rat.

Figure 5B shows the reaction of the conventional controller, PD controller with Peterka’s gain parameters (Peterka, 2002) and a non-linear PD controller for standing rats (Funato et al., 2021). Both models could stabilize the body after floor disturbances, but the behaviors were slightly different from the experiment. When we focus on the control torque τ, the control torque of every controller gradually increases after the floor torque τ_*Floor*_ increased, and the control torque τ becomes larger than the floor torque τ_*Floor*_. The disturbance *via* floor torque τ_*Floor*_terminates when time = 0.2 s and the CoM angle gradually returns to the vertical position. Here, the controllers react differently to the termination of the disturbance. The MPC controller smoothly reacted to the change in the floor torque τ_*Floor*_, while conventional controllers drastically changed the control torque.

Next, we investigated the features of the proposed model to reproduce the behavior of the rats. The proposed model has two MPC parameters, that is, the prediction horizon *H_p* and control horizon *H_u*, and three parameters related to the body system, the MTP stiffness *k_P*, the MTP viscosity *k_D*, and the magnitude of the noise σ. The effect of the two MPC parameters on the behavior is shown in Figures 5C–G.

Figure 5C shows the CoM angle and control torque τ of the simulation with the prediction horizon *H_p* from 30 ms to 1,300 ms. Here, the system became unstable when the prediction horizon was lower than 30 ms and higher than 1,300 ms. Each line in Figure 5C is the result of the simulation at each prediction horizon (the average of ten simulations to reduce the CoM variation due to noise). The CoM and torque patterns with a different prediction horizon *H_p* were similar to each other, but there were differences in the peak values. Figure 5D shows the maximum CoM angle, and Figure 5E shows the maximum torque (Figures 5D,E show the result of one sequence (Rat 1 Sequence 1), and Supplementary Figure 2 shows the result of whole sequences). These figures indicate that the maximum CoM decreases while the maximum torque decreases as the prediction horizon *H_p* increases. In other words, as the prediction horizon increases, the motion is designed to suppress the fluctuation in the CoM with less torque. This characteristic of prediction horizon *H_p* was consistent for 16 of 18 sequences (see also Supplementary Figure 2).

Following this, the control horizon *H_u* was varied from 2 ms to 50 ms, as shown in Figure 5F. Each line in the figure represents the simulation result according to each control horizon *H_u* (the average of ten simulations to reduce the CoM variation due to noise). Subsequently, the pattern changes gradually with increasing *H_u* values, and the fluctuation in the CoM and maximum torque become smaller with higher control horizon *H_u*. Figure 5G shows the correlation coefficient (cosine correlation) between the time series of the CoM angle with a control horizon of 50 ms and the time series of the CoM angle with each control horizon *H_u* (Figure 5G shows the result of one sequence (Rat 1 Sequence 1), and Supplementary Figure 3 shows the result of whole sequences). Figure 5G shows that the change in the time series of the CoM angle is obvious up to 20 ms, and becomes less obvious after 30 ms. This characteristic of control horizon *H_u* was consistent for all sequences (see also Supplementary Figure 3).

In summary, the longer control and prediction horizons suppressed the variation in the CoM angle with a smaller torque. Furthermore, the prediction horizon *H_p* affected the peak value, whereas the control horizon *H_u* affected the pattern. Based on the characteristics of these control parameters, we identified five unknown parameters.

### Identification Results of the Prediction Behavior

We searched for model parameters (prediction horizon *H_p*, control horizon *H_u*, MTP stiffness *k_P*, MTP viscosity *k_D*, and noise magnitude σ) whose simulation reproduces the measured time series of the rat’s CoM angle. Figure 6 shows the results, and the time series of the simulation with these parameters is shown in Figure 5A. The significance of the identified parameters was tested using a two-way ANOVA with the parameters and experimental sequences. The results did not show significant differences among experimental sequences (*P* = 0.47, f = 1.00, df = 17), but showed significant differences with the parameters (*P* < 0.001, f = 474.0, df = 4), implying the robustness of the identification results to different experimental sequences.

The top row of Figure 6 shows the MPC parameters. The mean (SD) of the result of the prediction horizon was 0.96 (± 0.19) s. This result indicates that the postural control can be predicted up to 0.96 s prior to the state. In the experiment, the rats received a light stimulus 0.9 s before the tilt disturbance of 0.25 s. The interval time between the sensory input and the disturbance was 0.9–1.15 s, which is close to the identified length of the prediction horizon. The mean (SD) of the result of the control horizon was 24.4 (± 8.6) ms. This result indicates that the prediction of the control input could change for 24.4 ms. Simulations with different control horizons (Figures 5F,G) showed that the CoM angle hardly changed when the control horizon was greater than 20 ms. This indicates that the identified control horizon is long enough to produce sufficiently complex inputs.

The lower portion of Figure 6 shows the parameters related to the body model. The mean (SD) value of the MTP stiffness *k_P* was 0.34 (± 0.13) *mgh*. This value is normalized with body mass (*m)*, acceleration of gravity (*g)*, and the length from the MTP joint to the CoM (*h).* Previous studies showed that ankle stiffnesses in humans to be approximately 0.3 *mgh* (Hof, 1998) to approximately 0.7 *mgh* (Morasso and Sanguineti, 2002). The identified value was almost comparable, which supports the validity of the identification results. The mean (SD) value of the MTP viscosity *k_D* was 0.03 (± 0.02) *mgh*. Previous studies (Asai et al., 2009; Suzuki et al., 2012) have assumed that the ankle viscosity is less than a tenth of the stiffness. The mean (SD) value of the noise magnitude σ was 0.50 (± 0.23) mNm. This value was in close agreement with the previously identified noise magnitude in the quiet standing rats (Funato et al., 2021).

## Discussion

In this study, we constructed a floor-tilting task for upright standing rats and compared these behaviors with a simulation that was based on MPC. In the experiment, the postural response of the rats due to disturbance dramatically reduced after experiencing 70 sequential trials, indicating that they acquired predictive control against the disturbance. The simulation showed that prolonging the predictive and control horizons allowed a reduction in the fluctuation of the CoM and peak control torque. The predictive horizon identified to match the simulation with the measured CoM data was close to the interval time, from the light turning off to the disturbance, suggesting that the rats predicted posture dynamics under the disturbance based on the timing of the sensory input.

During the initial trials in the experiment, the foot and leg segments rotated backward, contributing to increase in the CoM angle (Figures 3, 4). Similarly, in the bipedal standing of humans, the backward tilt of the floor displaces the CoP backward and rotates the shank backward (Diener et al., 1992; Carpenter et al., 1999; Kolb et al., 2002; Campbell et al., 2009). Regarding the movement of the segments above the trunk, the lower trunk did not significantly rotate on average, but the upper trunk and head rotated forward (Figure 4B). In human studies, the trunk is simplified into one segment. However, as it has been reported to rotate forward with the backward tilt of floors (Carpenter et al., 1999), the overall direction of rotation is consistent with this study. In rodents, the mass and length of the trunk account for greater proportions of the whole body than in humans (Table 1; Winter, 2009), and the trunk is not adapted to maintain upright positions as in humans. These characteristics seem to have led to the bending of the trunk as a result of the disturbance (Figure 3A). In any case, the overall mechanics by which a floor rotation evokes postural response is by the transmission of the rotation to the legs, giving backward momentum to the pelvis and lower trunk. This is common between bipedal humans and rodents.

The experience of 70 sequential disturbance trials dramatically reduced postural response, including backward rotation of the foot and the leg segments (Figures 3, 4). These results indicate that flexing of the ankle and the MTP joints at proper timing and amplitudes according to the floor tilt contributed to a reduction in the response. During the tilting tasks for humans, the entire sole was in contact with the floor, and the joints above the ankle determined the postural movement. On the other hand, it seems relatively easy for rodents to tiptoe on the hindlimbs (Funato et al., 2017), indicating that movements of the MTP joints can be significantly involved in postural control. Therefore, we used an inverted pendulum model around the MTP joint in this simulation. In humans, under the association of tilting disturbance and preceding sensory input, fluctuation of activities immediately before the tilting, suppression of stretch reflexes, and automatic postural response with a latency of approximately 100 ms are evoked at muscles around the ankle, knee, and hip joints (Kolb et al., 2002; Campbell et al., 2009). Although the present study is limited to measuring kinematics, similar adjustments are likely to be evoked in the muscles around the MTP joints.

We modeled the behavior of predictive postural control of rats using MPC. The postural control system of quiet standing has been modeled using feedback control such as proportional-integral-derivative (PID) control (Peterka, 2000, 2002) or intermittent control considering the dead zone of the sensory system (Asai et al., 2009; Gawthrop et al., 2014). The bipedal standing of the rats was also modeled using PID-based feedback control. To model the prediction of disturbances, the control needs to include a prediction mechanism. However, the postural control model with prediction has hardly been discussed. One study adopted MPC to model human postural control from the viewpoint of optimal control (Yao and Levine, 2009) and showed the time correlation of the body sway (estimated by Stabilogram-Diffusion Function) and the amplitude of the body sway were reproduced by the model with MPC. More recent research used MPC to model arm movement during a standing position and showed the contribution of the arm to achieve stabilization (Shen et al., 2021). MPC consists of the prediction of future states based on internal models and the optimization of the predicted states. Researches have demonstrated that the internal forward model (Miall and Wolpert, 1996) can be produced as the activity of Purkinje cells (Laurens et al., 2013; Herzfeld and Shadmehr, 2014), thus proving that prediction of the state could be possible. It is also possible to control optimization in the nervous system (Scott, 2004; Todorov, 2004). Therefore, the nervous system is equipped with a mechanism that enables MPC, and it is reasonable to model postural control with prediction using MPC. Our simulation results successfully reproduced the predictive behavior of rats, and the identification results for the MPC parameters were consistent with the experimental conditions and past identification results for postural control.

One of the advantages of our MPC model over the conventional PID-based feedback control models is that our model explicitly considers the internal model for prediction. This enables us to discuss the effect of learning the internal model on posture control. However, at the same time, flexibility in the internal model causes uncertainty. The internal model is composed of learning; thus, it could cause a large uncertainty, particularly during the learning process. Only one point that can determine the property of the internal model is after learning. After complete learning, the internal model is assumed to be a good copy of the actual body and environment. Therefore, in this study, we used them as an internal model. As a limitation, this system model can simulate only the behavior of rats after learning, and thus, we compared simulation results with the experimental results after learning. This model minimizes the uncertainty in the internal model and succeeds in the discussion of prediction control. However, the learning process in rats could not be discussed using the current model. To overcome this limitation, the internal model should include learning. The internal model used in our model is based on the equation of motion of the body and environment, and these equations can be learned by machine learning. Therefore, by including machine learning in our model, approaching the learning mechanism of rats using the model will become possible.

The interval time between the sensory input of light and the floor tilting was set within the range, allowing classical conditioning (Kolb et al., 2002) and our simulation results support the establishment of these associations. However, non-associative processes, such as habituation or adaptation, may also have contributed to the reduction in the amplitude of postural response due to disturbance (Nashner, 1976; Kolb et al., 2002, 2004). It will be important to experimentally distinguish between associative and non-associative learning in the future. The first strategy is to focus on preparatory activities before the disturbance (Kolb et al., 2002). In the experiment, a rat trembled in the interval time of several trials in the middle of learning (data not shown), suggesting that an association was about to be established. As a previous study pointed out that movement before floor tilt can be disadvantageous to postural stabilization (Kolb et al., 2002), it may be difficult to detect preparatory movements before disturbance at a kinematic level. However, examining muscle activities in antagonist muscles may capture these activities. The second strategy was to conduct control experiments. Experimental conditions without sensory input would quantify the rate of learning that is based on only non-associative learning, and conversely, giving sensory input alone under association would induce canceling movements at the time of the disturbance, which is based on association (Clark et al., 2002; Campbell et al., 2009). These refinements in experiments will play an important role in neuroscientific research on predictive postural control in the future. That is, the inactivation of specific areas in the nervous system clarifies the responsibilities for associative and non-associative learning, and identifying neural activities synchronizing with sensory input, disturbance, and muscle activities will help understand the coding and processing mechanisms of predictive postural controls in the nervous system.

## Data Availability Statement

The raw data supporting the conclusions of this article will be made available by the authors, without undue reservation.

## Ethics Statement

The animal study was reviewed and approved by the Ethical Committee for Animal Experiments at the University of Tokyo.

## Author Contributions

AK, DY, YM, and AF designed and conducted the experiment. TF, YM, and AF designed and executed the simulation. RS contributed to programming for the simulation. DY and TF conceptualized and supervised the whole study. AK, TF, and DY wrote the manuscript. All authors reviewed the manuscript.

## Conflict of Interest

The authors declare that the research was conducted in the absence of any commercial or financial relationships that could be construed as a potential conflict of interest.

## Publisher’s Note

All claims expressed in this article are solely those of the authors and do not necessarily represent those of their affiliated organizations, or those of the publisher, the editors and the reviewers. Any product that may be evaluated in this article, or claim that may be made by its manufacturer, is not guaranteed or endorsed by the publisher.
