# Supplementary material for: A Model of Predictive Postural Control Against Floor Tilting in Rats
Source: Front Syst Neurosci. 2021 Nov 25;15:785366. doi: 10.3389/fnsys.2021.785366 (PMC8655307; doi:10.3389/fnsys.2021.785366)
Supplement: Supplementary file 1 [file Data_Sheet_1.pdf]

frontiers

Supplementary Material

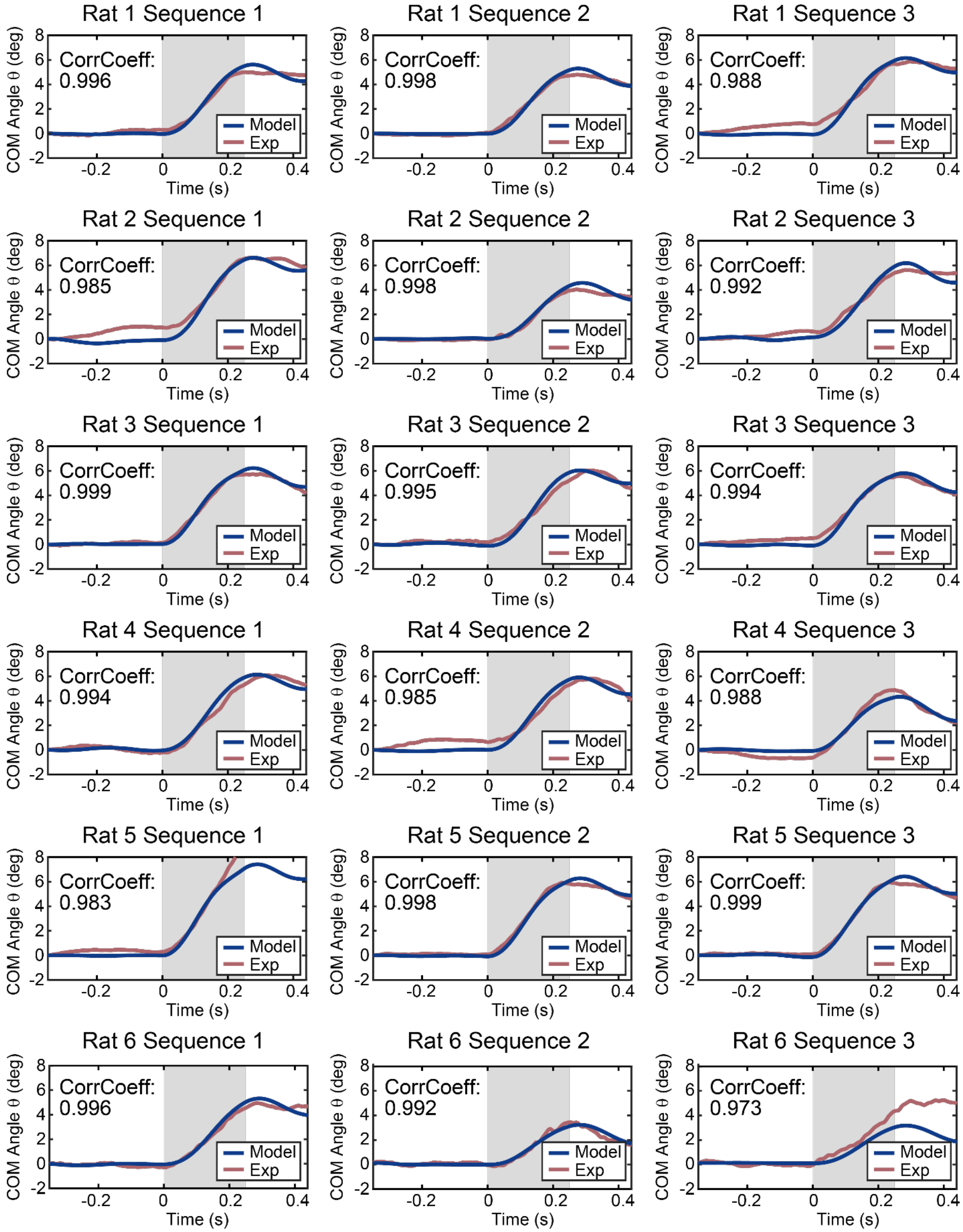

Supplementary Figure 1. Time series of the CoM angle of the simulation results of the model (Model) and the experimental results of the rats (Exp). Gray area represents the duration of floor tilting. Numbers with CorrCoeff are the correlation coefficient (cosine correlation) between the model (Model) and the experiment (Exp).

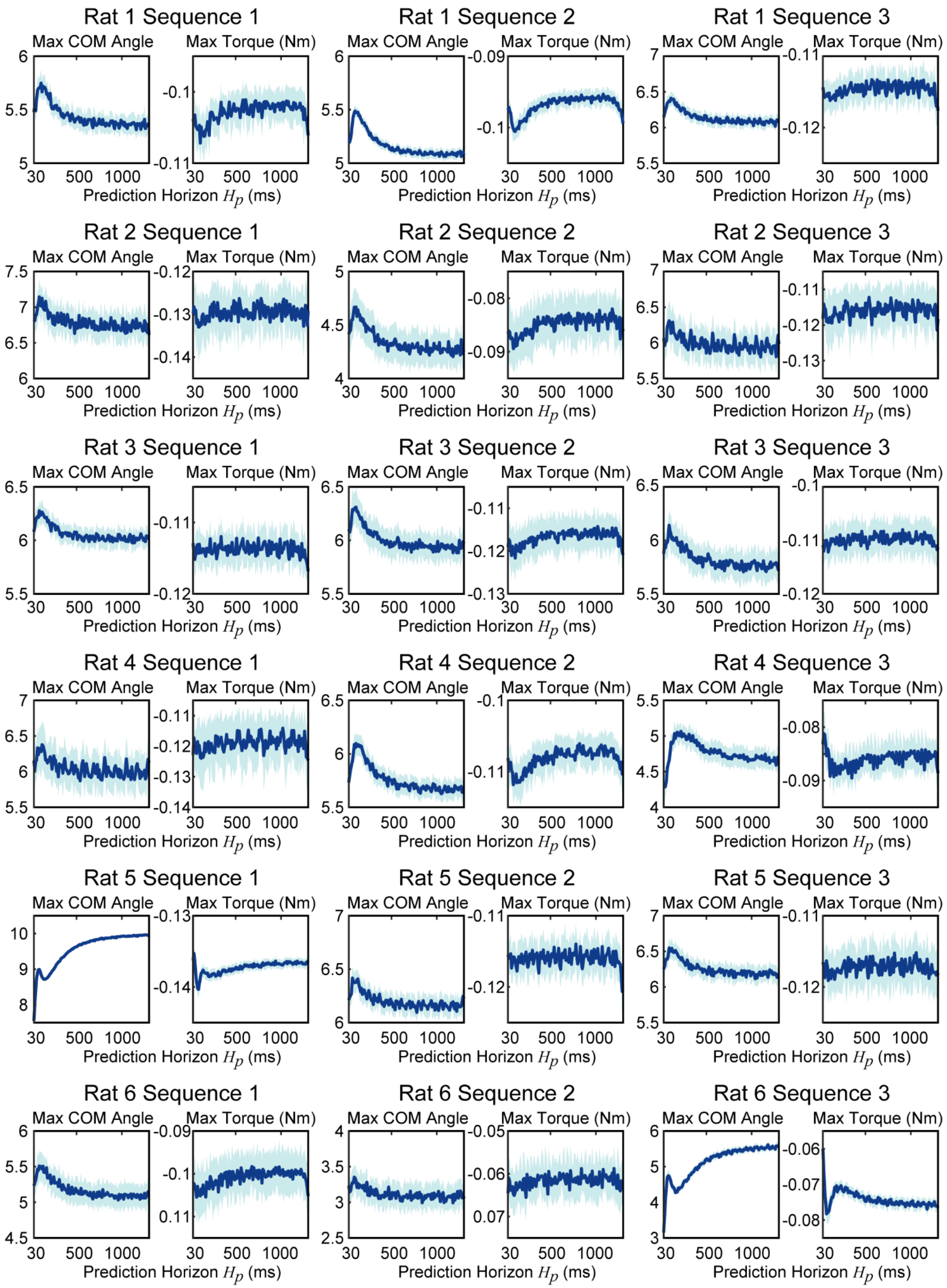
Rat 1 Sequence 1
Max COM Angle
6
5.5
5
Max Torque (Nm)
-0.1
-0.11
30 500 1000
Prediction Horizon H_p (ms)
Rat 1 Sequence 2
Max COM Angle
6
5.5
5
Max Torque (Nm)
-0.09
-0.1
30 500 1000
Prediction Horizon H_p (ms)
Rat 1 Sequence 3
Max COM Angle
7
6.5
6
5.5
Max Torque (Nm)
-0.11
-0.12
30 500 1000
Prediction Horizon H_p (ms)
Rat 2 Sequence 1
Max COM Angle
7.5
7
6.5
6
Max Torque (Nm)
-0.12
-0.13
-0.14
30 500 1000
Prediction Horizon H_p (ms)
Rat 2 Sequence 2
Max COM Angle
5
4.5
4
Max Torque (Nm)
-0.08
-0.09
30 500 1000
Prediction Horizon H_p (ms)
Rat 2 Sequence 3
Max COM Angle
7
6.5
6
5.5
Max Torque (Nm)
-0.11
-0.12
-0.13
30 500 1000
Prediction Horizon H_p (ms)
Rat 3 Sequence 1
Max COM Angle
6.5
6
5.5
Max Torque (Nm)
-0.11
-0.12
30 500 1000
Prediction Horizon H_p (ms)
Rat 3 Sequence 2
Max COM Angle
6.5
6
5.5
Max Torque (Nm)
-0.11
-0.12
-0.13
30 500 1000
Prediction Horizon H_p (ms)
Rat 3 Sequence 3
Max COM Angle
6.5
6
5.5
Max Torque (Nm)
-0.1
-0.11
-0.12
30 500 1000
Prediction Horizon H_p (ms)
Rat 4 Sequence 1
Max COM Angle
7
6.5
6
5.5
Max Torque (Nm)
-0.11
-0.12
-0.13
-0.14
30 500 1000
Prediction Horizon H_p (ms)
Rat 4 Sequence 2
Max COM Angle
6.5
6
5.5
Max Torque (Nm)
-0.1
-0.11
30 500 1000
Prediction Horizon H_p (ms)
Rat 4 Sequence 3
Max COM Angle
5.5
5
4.5
4
Max Torque (Nm)
-0.08
-0.09
30 500 1000
Prediction Horizon H_p (ms)
Rat 5 Sequence 1
Max COM Angle
10
9
8
Max Torque (Nm)
-0.13
-0.14
30 500 1000
Prediction Horizon H_p (ms)
Rat 5 Sequence 2
Max COM Angle
7
6.5
6
Max Torque (Nm)
-0.11
-0.12
30 500 1000
Prediction Horizon H_p (ms)
Rat 5 Sequence 3
Max COM Angle
7
6.5
6
5.5
Max Torque (Nm)
-0.11
-0.12
30 500 1000
Prediction Horizon H_p (ms)
Rat 6 Sequence 1
Max COM Angle
6
5.5
5
4.5
Max Torque (Nm)
-0.09
-0.1
0.11
30 500 1000
Prediction Horizon H_p (ms)
Rat 6 Sequence 2
Max COM Angle
4
3.5
3
2.5
Max Torque (Nm)
-0.05
-0.06
0.07
30 500 1000
Prediction Horizon H_p (ms)
Rat 6 Sequence 3
Max COM Angle
6
5
4
3
Max Torque (Nm)
-0.06
-0.07
-0.08
30 500 1000
Prediction Horizon H_p (ms)

Supplementary Figure 2. The maximum CoM angle and the maximum torque of the simulation results with a prediction horizon from 30 ms to 1300 ms. Blue lines show the average of ten simulation results with same parameters, and blue areas show their standard deviation. Note that the patterns have similar tendency except for Rat 5 Sequence 1 and Rat 6 Sequence 3.

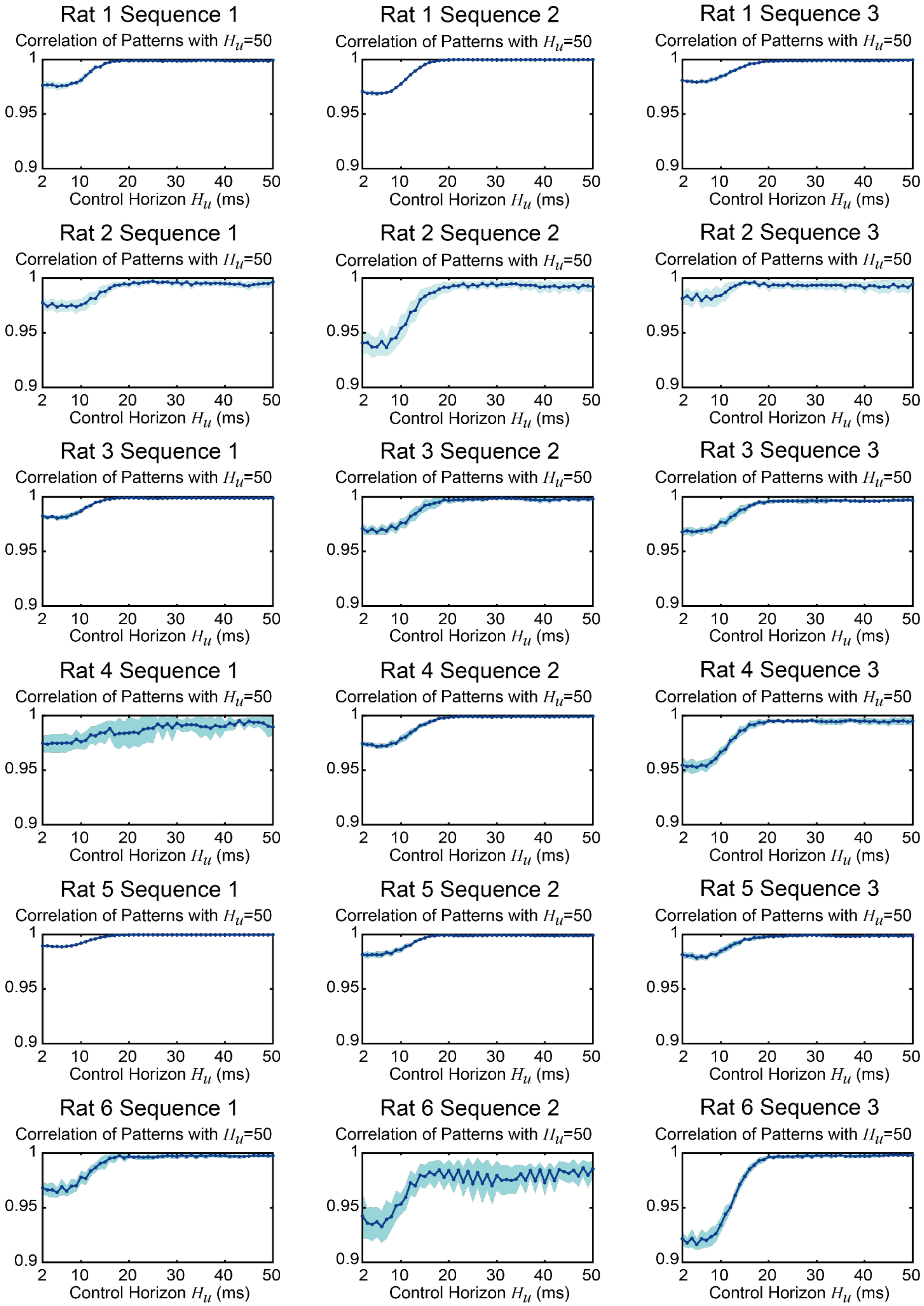
Rat 1 Sequence 1
Correlation of Patterns with Hu=50
Control Horizon Hu (ms)
Rat 1 Sequence 2
Correlation of Patterns with Hu=50
Control Horizon Hu (ms)
Rat 1 Sequence 3
Correlation of Patterns with Hu=50
Control Horizon Hu (ms)
Rat 2 Sequence 1
Correlation of Patterns with Hu=50
Control Horizon Hu (ms)
Rat 2 Sequence 2
Correlation of Patterns with Hu=50
Control Horizon Hu (ms)
Rat 2 Sequence 3
Correlation of Patterns with Hu=50
Control Horizon Hu (ms)
Rat 3 Sequence 1
Correlation of Patterns with Hu=50
Control Horizon Hu (ms)
Rat 3 Sequence 2
Correlation of Patterns with Hu=50
Control Horizon Hu (ms)
Rat 3 Sequence 3
Correlation of Patterns with Hu=50
Control Horizon Hu (ms)
Rat 4 Sequence 1
Correlation of Patterns with Hu=50
Control Horizon Hu (ms)
Rat 4 Sequence 2
Correlation of Patterns with Hu=50
Control Horizon Hu (ms)
Rat 4 Sequence 3
Correlation of Patterns with Hu=50
Control Horizon Hu (ms)
Rat 5 Sequence 1
Correlation of Patterns with Hu=50
Control Horizon Hu (ms)
Rat 5 Sequence 2
Correlation of Patterns with Hu=50
Control Horizon Hu (ms)
Rat 5 Sequence 3
Correlation of Patterns with Hu=50
Control Horizon Hu (ms)
Rat 6 Sequence 1
Correlation of Patterns with Hu=50
Control Horizon Hu (ms)
Rat 6 Sequence 2
Correlation of Patterns with Hu=50
Control Horizon Hu (ms)
Rat 6 Sequence 3
Correlation of Patterns with Hu=50
Control Horizon Hu (ms)

Supplementary Figure 3. The correlation coefficient (cosine correlation) between the time series of the CoM angle with control horizon 50 ms and the time series of the CoM angle with each control horizon. Blue lines show the average of ten simulation results with same parameters, and blue areas show their standard deviation.
